# The effect of SODIS water treatment intervention at the household level in reducing diarrheal incidence among children under 5 years of age: a cluster randomized controlled trial in Dabat district, northwest Ethiopia

**DOI:** 10.1186/s13063-018-2797-y

**Published:** 2018-07-31

**Authors:** Bikes Destaw Bitew, Yigzaw Kebede Gete, Gashaw Andargie Biks, Takele Tadesse Adafrie

**Affiliations:** 1 Department of Environmental and Occupational Health and Safety, Institute of Public Health, College of Medicine and Health Sciences, University of Gondar, Gondar, Ethiopia; 2 Department of Epidemiology and Biostatistics, Institute of Public Health, College of Medicine and Health Sciences, University of Gondar, Gondar, Ethiopia; 3 Department of Health Service Management and Health Economics, Institute of Public Health, College of Medicine and Health Sciences, University of Gondar, Gondar, Ethiopia; 4 Department of Epidemiology and Biostatistics, School of Public Health, College of Health Sciences and Medicine Referral Hospital, Wolaita Sodo University, Wolaita, Ethiopia

**Keywords:** Under-five children, Diarrhea, SODIS, RCTs, Northwest Ethiopia

## Abstract

**Background:**

Solar Disinfection (SODIS) of water is an economical, user-friendly, and environmentally safe household water treatment method that has been advocated as a means of decreasing the burden of diarrhea among children under 5 years of age. Laboratory studies have consistently shown the efficacy of the SODIS method to destroy waterborne pathogens. However, the evidence-based health effect of a SODIS intervention at the household level is limited. The main aim of the study was to examine the effectiveness of a SODIS intervention in reducing the incidence of diarrhea among under-five children.

**Methods:**

A community-based, cluster randomized controlled trial was conducted, over 6 months from 10 January to 7 July 2016, in 28 rural villages of northwest Ethiopia. In the intervention group, 384 children in 279 households received polyethylene terephthalate (PET) bottles, and in the control group 394 children in 289 households who continued to use their usual drinking-water sources were included in the trial. The study compared diarrheal incidence among the intervention group children who were exposed to SODIS household water treatment and the control group children who were not exposed to such water treatment. A generalized estimating equation (GEE) model was used to compute the adjusted incidence rate ratio and the corresponding 95% confidence interval.

**Results:**

In this trial, the overall SODIS compliance was 90.6%. The incidence of diarrhea was 8.3 episodes/100 person-week observations in the intervention group compared to 15.3 episodes/100 person-week observations in the control group. A statistically significant reduction was observed in the incidence of diarrhea in the intervention group compared to the control (adjusted IRR 0.60 (95% CI 0.52, 0.70) with a corresponding prevention of 40% (95% CI: 34, 48).

**Conclusion:**

The SODIS intervention substantially reduced the incidence of diarrhea among under-five children in a rural community of northwest Ethiopia. This indicates that a SODIS intervention is an invaluable strategy that needs to be integrated with the National Health Extension Program to be addressed to rural communities.

**Trial registration:**

Clinical Trial Registry India, ID: CTRI/2017/09/009640. Registered retrospectively on 5 September 2017.

**Electronic supplementary material:**

The online version of this article (10.1186/s13063-018-2797-y) contains supplementary material, which is available to authorized users.

## Background

Waterborne diarrheal diseases are prevalent globally in areas where drinking-water treatment is inadequate [1]. The consumption of contaminated water, inadequate sanitation and hygiene are the main (88%) contributors to an estimated four billion cases of diarrhea every year, causing 1.8 million deaths, out of which about 90% are children under 5 years of age [2, 3]. The magnitude of diarrheal disease, due to which an estimated 801,000 children aged under 5 years die each year, is high in developing countries [4, 5].

The consumption of water from unimproved sources is a potential contributor to diarrheal diseases [6]. Though the Millennium Development Goal (MDG) of safe drinking-water has been globally achieved, 663 million people still relied on unimproved water sources in 2015. In sub-Saharan Africa where safe water coverage is less than 50%, about 319 million people lack access to improved water sources [7].

In Ethiopia, increasing household access to safe drinking-water is a long-standing development goal adopted by the government over the past two decades [8]. According to WHO/UNICEF 2015 Joint Monitoring program (JMP) country report, the coverage of improved water source was 93% among the urban population of Ethiopia, 49% in rural areas, and 57% at the national level [7]. But still nearly 40 million Ethiopians, most of them in rural areas, do not have access to safe drinking-water [7, 9]. Moreover, more than 90% of households do not treat their drinking-water at home [8]. Such a situation would pose high public health risks to unsafe water users unless prompt intervention is implemented [10]. According to the 2016 Demographic and Health Survey of Ethiopia (2016 EDHS) the under-five mortality rate in the country was 67 per 1000 live births, and about 11% deaths occurred due to diarrhea per year [8, 11] .

Studies conducted in different parts of Ethiopia indicated that a 2-week prevalence of diarrhea among under-five children (U5C) ranged from 22.5 to 30.5% [12–14]. The risk factors for diarrheal incidence as revealed by various studies were lack of a piped water supply [15–18], poor water-handling practices [16, 17, 19], lack of water treatment at home [20], types of water container [20], poor sanitation [14, 21, 22], lack of hand-washing facilities [14, 22], living in rural areas [14], child age [14, 19], and low socioeconomic status [15, 22].

The Amhara Region is one of the administrative areas in Ethiopia that has faced water-linked, life-threatening challenges for many years [23]. The accessibility of any of the alternative household water treatment (HWT) methods is still limited, and some, such as Solar Disinfection (SODIS), are unknown. Due to this, most of the people in the study area were consuming untreated water exposing them to a high risk for diarrheal diseases. For example, the magnitude of diarrhea among U5C was reported as 21.5% at a rural district level [23, 24].

The installation of large-scale water treatment plants in rural Ethiopia is difficult due to the scarcity of resources and scattered settlement [25]. Therefore, the situation demands the implementation of alternative strategies which are easily applicable, low-cost, and environmentally friendly HWTs [26]. HWTS can be more effective in preventing diarrhea than centralized water treatment [27]. A comprehensive Cochrane review of controlled trials in 19 countries revealed that household-based water treatment interventions were about twice as effective in preventing diarrheal disease (47%) than improved water sources (27%) [28]. Household water treatment and safe storage is one of the seven strategies suggested by the WHO and UNICEF for the prevention of diarrhea among children [29]. Moreover, it is also the priority area of the current national drinking-water-quality monitoring to ensure drinking-water safety to reduce waterborne diseases in Ethiopia [30].

Solar water disinfection (SODIS) where raw water is filled into polyethylene terephthalate (PET) bottles is one of the potential alternative HWT technologies that relies on the germicidal effects of sunlight and heat. Most of rural peoples in Ethiopia are not aware that SODIS is used as a HWT method [31]; whereas, over five million people in more than 50 developing countries disinfect their regular drinking-water by using SODIS [32]. Today, laboratory studies have consistently shown the efficiency of SODIS in significantly reducing the risk of a microbial contamination of drinking-water [33, 34]. Though, an evidence-based health effect of SODIS intervention at the household level is limited, diarrheal disease problems could be prevented through improving the quality of drinking-water [35]. SODIS as a HWT method is one particular option within the broader water safety plan to emphasize the value of preventive health intervention; it provides the greatest benefits to young children who are most likely to suffer from diarrhea [35]. Various studies [36–40] have revealed safety evidences that no harmful byproducts, such as plasticizers, were found to be higher than WHO limits in drinking-water in the sunlight-exposed PET bottles that were reused for over a period of 6 months. So, this useful value could be researched through community trials. Therefore, one of the primary aims of this interventionist study was to examine the effect of SODIS water treatment in reducing diarrheal incidence among U5C using a cluster randomized controlled trial (RCT) design in a rural community of northwest Ethiopia.

## Methods

### Study settings and population

**Fig. 1:**
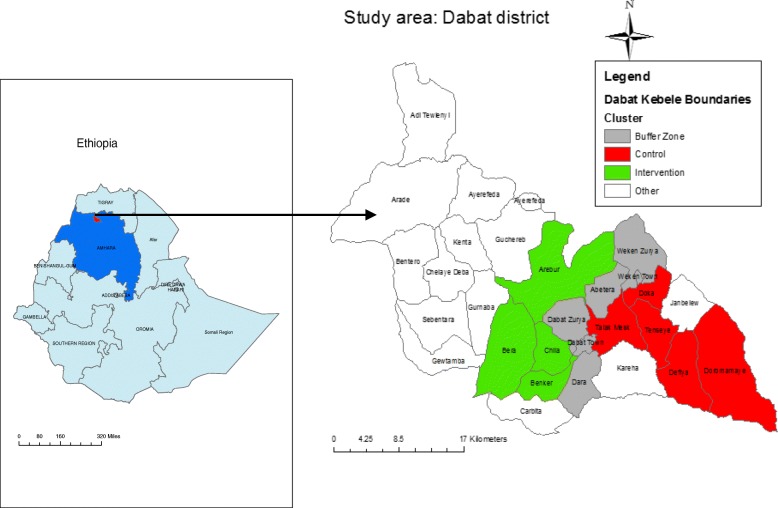
Map of Ethiopia with Amhara Administrative Region, Dabat district and Dabat HDSS site with “green” as demarcation color of study areas for intervention, “red” for the control group and “gray” for the buffer zones, 2016

The study was conducted in Dabat district, northwest Ethiopia (Fig. 1). In 1996, the University of Gondar selected Dabat district to serve as a site for the Health and Demographic Surveillance System (HDSS), and established the Dabat Health Research Center (DRC) which is one of the six HDSS sites in Ethiopia. The site runs its surveillance in four urban and nine rural *kebeles* (lowest local administration units in Ethiopia). Dabat district has three geo-climatic zones, namely “*Dega*” (highland and cold), “*Woina Dega*” (mid land and temperate) and “*Kolla*” (low land and hot). An all weather road runs from Gondar city through Dabat to some towns in the Semien National Park and Tigray Region. The total population of the HDSS site was estimated at about 70,611 by a 2012/2013 projection. The ratio of male to female inhabitants was almost 1:1. The number of children under 5 years of age was 7918. Out of the total population in the study area, 53,482 (75.74%) were from rural areas. Each of the total 6314 households had at least one child under 5 years of age [23]. Almost all rural households collect water from wells, springs, or streams/ rivers and store their drinking-water in 20-L Jerry cans with tight covers [31].

### Trial design

A community-based, cluster randomized controlled, parallel-group trial was conducted in Dabat district to examine the effect of SODIS water treatment on childhood diarrhea reduction from 10 January to 7 July 2016.

### Sample size and sampling method

Sample size was determined using methodological approaches published by Hayes and Bennett [41] and assuming a 10.4% incidence of diarrhea among children in the control group based on a previous study [42]. According to a practical guide to cluster randomized trials in health service research, intracluster coefficient (ICC) is a convenient way to estimate cluster variability. Eight pairs of clusters were required to detect a difference of at least 32% in the incidence rate between the control and intervention groups with 80% power, ICC = 0.02, 95% confidence interval with a 5% alpha level, and design effect [1 + (m − 1)ICC] of 1.6 from clustering. Anticipating a dropout of at least one cluster per group and a loss of follow-up of individuals, the allocation ratio was almost 1:1. The final sample size was adjusted to 14 pairs of cluster (28 clusters) with average size (*m*) of 30 children per cluster (a village), followed for 12 weeks (which can provide 5040 person-week observations in each group). After identifying families that did not use any water treatment methods using a baseline survey, households with U5C that fulfilled the selection criteria were included in the cluster RCT schemes.

### Cluster selection

The cluster selection criteria were (1) geographical accessibility of cluster areas throughout the year, (2) average cluster size of 28–30 children aged under 5 years, (3) reliance only on untreated drinking-water sources, and (4) unavailability of other special water-quality management interventions and diarrheal disease prevention programs in the community.

The selection process of eligible clusters (villages) was as follows. Initially, the study area was divided into two blocks/arms (A and B) with a buffer zone between them. This was done to minimize cross-contamination of information. Potential clusters in the study area were listed and sorted out based on the preset selection criteria within the two blocks. Under the three geo-climatic zones, “Dega,” “Woina-Dega,” and “Kolla” cluster/village 30, 12, and 21 were grouped, respectively.

Intervention and control arms were randomly assigned by the lottery method. Each cluster code was written on a piece of paper and put on a table after being rolled in the presence of the Dabat HDSS site and *woreda* administrative staff who helped to maintain transparency and avoid selection biases. The agreement criteria were established before the lottery was cast to reach a consensus that the first lot picked would be the intervention block. One of the *woreda* administrators drew the lots and read the results loudly to the audience throughout the selection process. For assuring the appropriateness of the procedures, the other lots were drawn checked. Clusters in each arm were selected by the simple random sampling technique using the lottery method. Fourteen clusters (villages) were randomly picked out of 31 clusters from the intervention arm and 14 clusters out of 32 in the control arm. In order to minimize the effects of geo-climatic variations, the distribution of clusters between the intervention and control groups in each of the climatic zones was equally allocated. Accordingly, the pair of six clusters from Dega, three clusters from Woina-Dega, and five clusters from Kolla were selected randomly after stratification of the clusters by climatic zones. On average, 20 households with at least one child of 6–59 months of age were randomly selected in each cluster. Nineteen data collectors and six supervisors approached the mother-child pairs in the selected households and completed the baseline survey and the follow-up study. In addition, 11 local persons who lived in the same villages of intervention were recruited for the purpose of occasionally reminding household members to properly handle the PET bottles, expose water-filled bottles to sunlight in time, and monitor the implementation of the SODIS procedures.

### Measurement and definition

Exposure variable: households with U5C who were offered SODIS bottles and agreed to use them for drinking-water treatment by exposing them to direct sunlight radiation were defined as the “exposed category” of the study subjects. Households with U5C that were not offered SODIS bottles for drinking-water treatment were defined as the “unexposed category.”

The occurrence of diarrhea was used as the primary outcome measure to estimate its risk. The national perspective of diarrhea is similar to the WHO definition: “three or more loose or watery stools over the past 24 h (or more frequent than it is normal for the individual)” [29]. The incidence of diarrhea was computed as the number of U5C who had new episodes of diarrheal disease within 6 months of the follow-up period divided by the total number of person-week observations in the same period [43]. A new episode of diarrhea meant the occurrence of diarrhea after a period of three diarrhea free-days [44].

**Fig. 2:**
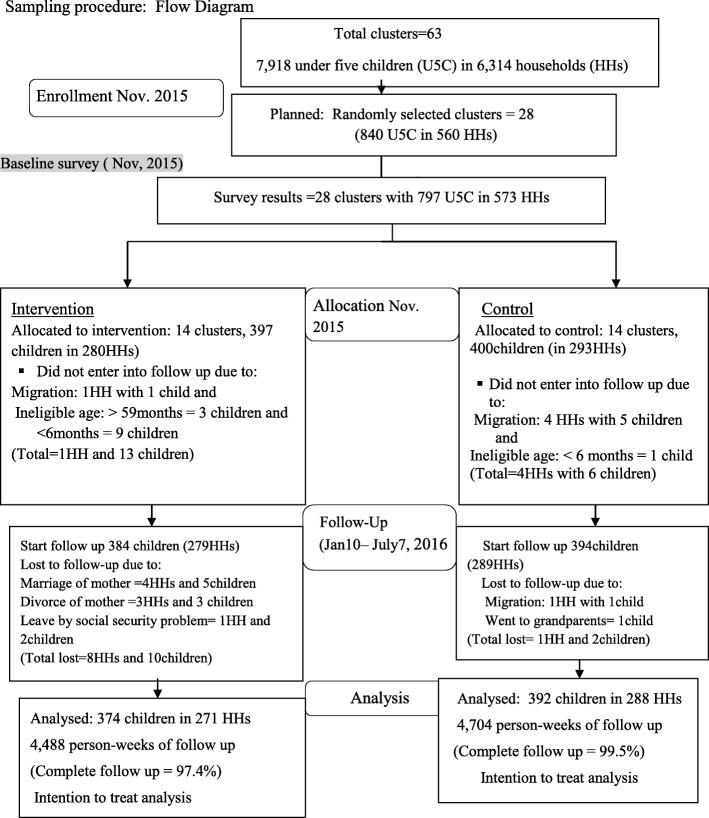
Sampling procedure for the cluster randomized controlled trial flow diagram on solar water disinfection (SODIS) at household level in Dabat district, northwest Ethiopia, 2016

### Data collection tools and procedures

Prior to the intervention study, a baseline survey was conducted to assess demographic and socioeconomic conditions, environmental sanitation and associated behavioral factors, and pre-intervention diarrhea prevalence rates. When the intervention study was conducted, data were collected about the incidence of diarrheal disease in both the intervention and control groups and compliance to SODIS procedures in the exposed category of the study households every 2 weeks during the follow-up period. Data were collected by using a structured questionnaire and a monthly pictorial diarrhea diary which consisted of a series of child-face pictures corresponding to daily diarrhea frequency that was simply filled daily by any responsible household members.

The questionnaire for baseline data collection was contextually prepared and modified based on WHO and UNICEF core questions on drinking-water and sanitation for household surveys. It consists of a set of harmonized questions widely used by many countries to make the data accurate and comparable across the globe. The structured and pre-tested questionnaire with a pictorial observational checklist, prepared after searching the academic literature [45, 46], was also used to collect follow-up data and information through face-to-face interviews of mothers/caregivers. Mothers’ identity numbers (including child ID and household code number) were prepared and given to the community by the Dabat Research Center at the University of Gondar.

### Water-quality analysis

Drinking-water-quality analysis was conducted to maintain periodic checkups of water source variations through a follow-up study. Water samples were collected from one fourth (25%) of the randomly selected households, water storage containers, and community water sources at the beginning and mid-point of the follow-up study period according to the standard methods described in the WHO guidelines for examining the quality of drinking-water [47]. Water samples from untreated and SODIS-treated water sources were collected in 500-mL sterilized glass bottles tightly closed, properly labeled, and transported in ice-packed cold boxes to the District Health Center Analytical Laboratory for bacteriological analysis. Samples were processed within 6 h using a membrane filtration technique (Millipore, Burlington, MA, USA) and cultured on membrane lauryl sulphate broth and pouring them into an absorbent pad (Oxoid Limited, Basingstoke, UK) at a 44.5 °C incubation temperature and the reading was taken within a 24-h growth period. *Escherichia coli*, the universally accepted indicator organism of fecal contamination of drinking-water, was determined (Coliform Forming Units (CFU) per 100-mL sample) [48, 49]. For quality control, a blank test with total coliforms at 37 °C was incubated in each process. Residual chlorine (mg/L) and turbidity in Nephelometric Turbidity Units (NTU) were also measured at sampling points and in the laboratory, respectively.

#### Intervention

The SODIS intervention was designed according to the Swiss Federal Institute of Aquatic Science and Technology (EAWAG) published guideline [50]. Each of the participant households in the intervention group was provided with six new PET bottles of 1- and 2-L capacity based on average family size, durable plastic containers for keeping the bottles, a piece of corrugated iron sheet (CIS), and instructional leaflets about the SODIS process for its daily implementation. The house-to-house campaign was the main strategy used to disseminate the method with demonstrations about the SODIS process by the data collectors and supervisors with the help of local persons. The mothers/caregivers were instructed to filter water with a clean cloth or local sieve after plain sedimentation if the water had visible turbidity, fill three fourths of each of the PET bottles with clear water, thoroughly shake the bottles to increase dissolved oxygen, and fill them fully, cover them with an air-tight lid, and place them horizontally on a reflective CIS in a pre-selected location that has received the maximum direct sunlight. Household members were also instructed to continuously expose the bottles to sunlight at least for 6 h from 09:00 am to 4:00 pm, and use the water for drinking after an overnight cooling. Instructions were also given to mothers/caregivers not to transfer the SODIS-treated water to other containers for preventing cross-contamination. In the intervention households, 1674 PET bottles were distributed and some reserve bottles were also secured for later substitutions of damaged or misused PET bottles.

### Control

The study households in the control group were not provided with SODIS bottles for water treatment except pictorial diary forms with pens. Households used the usual community water sources from wells, springs, or river water without the delivery of health education.

Compliance with the SODIS method was measured using four different indicators: (1) number of SODIS bottles exposed to sunlight, (2) number of bottles found ready to drink at home, (3) field worker’s direct observation at home and their subjective judgment (witness) about the status of the usage of SODIS bottles by households (user-status), and (4) bacteriological analysis of SODIS water at the start and middle of the follow-up period.

### Data quality control

The baseline and follow-up data collection tools were prepared in English after reviewing the academic literature. Then, they were contextually modified based on the concepts of the WHO and UNICEF as given in the “Core questions on drinking-water and sanitation for household surveys” [51]. The questionnaire was pretested and validated on primary caregivers who had similar characteristics as the study participants and lived near the study area. The questionnaire was translated from English to the local language, Amharic, for data collection and back to English for data entry purposes. The translation was done by the principal investigator and checked by language experts. Three days’ training was given to 19 data collectors and six supervisors based on the training manual for the improvement of data collection skills, and they were involved in the data collection processes under close supervision. Re-interviewing 5% of the households and rechecking the filled questionnaire on a daily basis, together with the feedbacks, were conducted by the supervisors. The data collectors and supervisors were randomly rotated among communities at the mid follow-up period. Assessments of the data clerk with frequent orientations and double entry of the data of 10% of the questionnaire were done to evaluate the mistakes likely to occur during data entry and data clearance practices. To maintain the quality of this RCT report, the Consolidated Standards of Reporting Trials (CONSORT) 2010 Checklist [52] was considered throughout the work.

### Statistical analysis

Data were entered into EPI INFO version 3.5.3 (CDC, Atlanta, GA, USA) and analyzed using STATA version 14.0 (StataCorp, College Station, TX, USA). Descriptive statistics were used to determine percentages, means, and standard deviations of the participants. A chi-square (*χ*^2^) test was applied to check the uniformity of baseline data between the intervention and control households. An intention-to-treat analysis was used to compare the incidence of diarrhea among children under 5 years of age between intervention and control groups. A generalized estimating equation (GEE) with a log-link Poisson distribution family and the exchange working correlation, with robust standard error (SE) estimation, was used for the analysis of repeated observations of diarrhea in individuals over time to consider the clustered nature of the data. The unadjusted and adjusted incidence rate ratio (IRR) along with the corresponding 95% confidence intervals (CI) was analyzed by using a multivariable analysis to control potential confounders.

## Results

### Characteristics of intervention and control groups

A total of 397 U5C in 280 households in the intervention and 400 U5C in 293 households in the control group were enrolled in 28 clusters (villages). The average size of a cluster was 28 children. The average number of participating households with U5C per cluster was 20. Five households and 19 children were not included in the follow-up study due to migration out of the study *kebeles* (Fig. 2). Nine (1.58%) households with 12 (1.54%) children were reported lost to follow-up. With all these exclusions, the data of 384 U5C (279 HHs) and 394 U5C (289 HHs) in the intervention and control groups, respectively, were finally entered into the follow-up scheme. In the 6-month follow-up study, data were collected about the occurrence of diarrhea among 9312 person-week observations with 97.4% and 99.5% follow-up completion rates in the intervention and control groups, respectively (Fig. 2). Table 1 illustrates the baseline socio-demographic and that the economic characteristics in the intervention and control households did not have significant differences (*p* > 0.05). The mean (± standard deviation (SD)) ages of the adult respondents and the U5C were 30.5(± 7.8) years and 29.3(± 15.5) months, respectively. The majority (83.9%) of mothers/caregivers had no formal education. The great majority (98.3%) of the heads of households were farmers. All of the houses (100%) had mud-flooring and most (69.6%) were roofed with CISs, while 64.7% sheltered domestic animals in the same premises. In the community, 67% of the households mainly got their drinking-water from unimproved sources. Springs, streams/rivers, and wells were the sources for, 45.2, 31.2, and 23.6% of the households, respectively. Very few (2.8%) households treated their drinking-water at the household level. There was no significant variation in the source of water and water treatment practices between the intervention and control groups at the baseline survey. Only 20.4% households had latrines. Most (92.1%) of the mothers/ caregivers washed their hands after handling waste. Before the start of the intervention study, a 2-week prevalence of childhood diarrhea was 20.8% in the intervention households and 21.8% in the control. In general, at baseline, the intervention and control villages had almost similar sanitation, hygiene, and water-handling practices (Table 1).

**Table 1:** Baseline characteristics of community and households of the randomized control trial, Dabat district, northwest Ethiopia, November 2015

Characteristics	Intervention *n* (%)	Control (%)	*p* value
Primary caregivers of children			
Mean age (± SD) (year)	30.7 (± 7.1)	30.2 (± 8.4)	0.08
No formal education of mother	242 (86.4)	239 (81.6)	0.11
Occupation (housewives)	279 (99.6)	291 (99.3)	0.23
No formal education of father	225 (80.4)	246 (84.0)	0.16
Occupation of heads of households (Farmer)	278 (99.3)	285 (97.3)	0.20
Economic indicators			
Own land	276 (98.6)	282 (96.2)	0.08
Own mobile	50 (17.9)	62 (21.2)	0.32
Own radio	23 (8.2)	28 (9.6)	0.57
Own livestock	275 (98.6)	282 (96.2)	0.45
Housing condition			
Corrugated iron sheet (CIS) roof	203 (72.5)	196 (66.9)	0.15
Separate kitchen	29 (10.4)	25 (8.5)	0.46
Live together with animals	198 (67.5)	182 (62.1)	0.18
Drinking-water			
Unimproved water source	186 (66.4)	198 (67.6)	0.77
Well	73 (26.2)	62 (21.2)	
Spring	141 (50.4)	118 (40.3)	
Stream/river	66 (23.6)	113 (38.6)	
Jerry can for home water storage	279 (99.6)	290 (99.0)	0.34
Treatment of drinking-water (any method)	6 (2.1)	10 (3.4)	0.36
Sanitation and hygiene			
Waste disposal (proper)	207 (73.9)	211 (77.1)	0.61
Functional latrine (proper)	34 (68.0)	54 (80.6)	0.12
Separate hand washing facility present	117 (41.8)	117 (39.9)	0.65
Hand washing agents (soap/ash)	57 (20.4)	51 (17.4)	0.37
Separate cup for child drinking	26 (9.3)	23 (7.8)	0.54
Hand-washing before child feeding	267 (95.4)	275 (93.3)	0.43
Hand-washing after toilet	199 (71.1)	229 (78.2)	0.051
Hand-washing after waste handling	259 (92.5)	269 (91.8)	0.76
Children characteristics			
Number of under-five children	397	400	0.86
Mean (± SD) age of children (months)	28.8 (± 15.9)	29.7 (± 15.1)	0.04
Male children	204 (51.4)	203 (50.8)	0.89
Measles vaccinated	291 (85.6)	305 (85.4)	0.95
Rota virus vaccinated	126 (31.7)	116 (29.3)	0.46
Two-week prevalence of diarrhea	80 (20.8)	86 (21.8)	0.74

### Intervention compliance

**Fig. 3:**
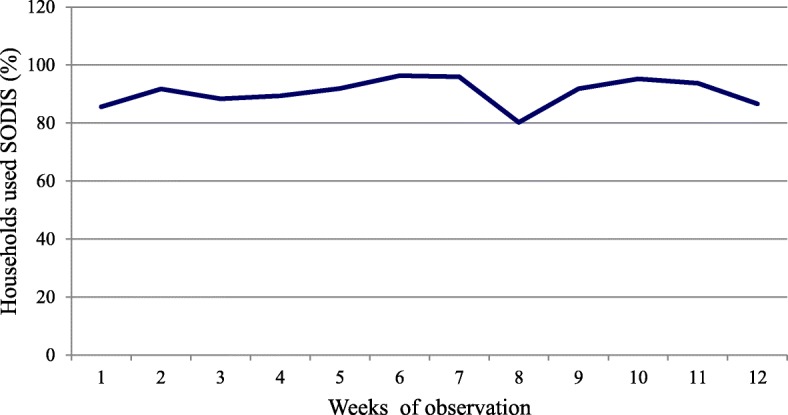
Percentage of SODIS user households during the 6-month observation period, Dabat district, northwest Ethiopia, 10 January to 7 July 2016

Seventy-six percent of the households in the intervention group exposed SODIS bottles to sunlight and 77% had SODIS bottles with ready-to-drink treated water. The judgment (witness) of field workers was rated that adherence to SODIS by 87.1% of the intervention households was good. The overall coverage of the SODIS implementation among the intervention households was 90.6%. In the remaining 9.4% of the intervention households, negligence and cloudy days were the two major reasons for not exposing SODIS bottles to sunlight. According to the field workers’ direct observation, 67.6% of the households exposed clean bottles filled with clear water to sunlight, 44% placed them on pieces of CIS, and 26.2% on rooftops. Mothers/caregivers were the most (in 92.6% of cases) responsible persons for managing SODIS process at home. Most of the households handled SODIS bottles properly, 75.7% of them using local shelves made of wood and mud and 21.7% storing in durable plastic containers. Very few households (2%) misused SODIS bottles for other domestic purposes, mostly for carrying edible oil or kerosene. Among the SODIS users, 74.4% U5C drank treated water directly from SODIS bottles (Table 2). Figure 3 illustrates the trends of households in maintaining the practice of SODIS water treatment during the 6-month follow-up period. During the eighth week of data collection, SODIS practice was down because of cloudy and rainy days.

**Table 2:** SODIS utilization among intervention households during the 6 months of 12 observations (*n* = 3261 household-weeks of observations) in Dabat district, northwest Ethiopia, 10 January to 7 July 2016

Characteristics	Number	Percent (%)
Regular SODIS practices		
Yes	2953	90.6
No	308	9.4
Reasons for non-exposure of SODIS bottle (*n* = 308)		
Negligence	121	39.3
Cloudy day	56	18.2
Work load	53	17.2
Bottle lost	3	1.0
Boiling	2	0.6
Other reasons	73	23.7
Observation of SODIS bottles (physical condition)		
Clean bottle filled with clear water	2205	67.6
Clean bottle filled with turbid water	282	8.6
Dirty bottle filled with clear water	21	0.6
Dirty bottle filled with turbid water	12	0.4
No observation bottles at the time of visits	741	22.7
Responsible person for SODIS process at home		
Mother	3020	92.6
All family members	149	4.6
Children	71	2.2
Father	21	0.6
SODIS bottle exposed to sunlight at visiting time		
Yes	2479	76.0
No	782	24.0
SODIS bottle with ready-to-drink water at the time of visiting		
Yes	2512	77.0
No	749	23.0
SODIS water users		
All family members	2261	69.3
Only U5C children	1000	30.7
U5C who drank untreated water during follow-up study period		
Yes	1196	36.7
No	2064	63.3
Means of drinking		
SODIS bottle	2426	74.4
Common cup with family	694	21.3
Separate cup for children	141	4.3
Type of surfaces of support for SODIS bottle		
Pieces of CISs laid on ground/bench top	1435	44.0
Roof top	853	26.2
Everywhere outside the house	839	25.7
Pieces of black plastic sheet	134	4.1
Reasons for shadow (*n* = 90)		
Tree shadow	66	73.3
Building shadow	24	26.7
Storing of SODIS bottle inside the house		
In shelf	2468	75.7
In durable plastic bag	708	21.7
Anywhere in the house	85	2.6
Use of SODIS bottle for other domestic purpose (*n* = 65)		
For cooking oil	32	49.2
For kerosene	23	35.4
For milk	3	4.6
For others	7	10.8
Observation of field workers about SODIS practices		
Good	2839	87.1
Poor	422	12.9

Legend: *CIS* corrugated iron sheet, *SODIS* solar disinfection, *U5C* under-five children

### Drinking-water quality

Out of the 245 water samples, 57 were taken from SODIS bottles and 188 from untreated water sources to analyze drinking-water quality both at control and intervention households. The overall results of bacteriological analysis of water samples from untreated community water sources showed that 90.7% of samples in the control and 91.2% in the intervention households were reported *E. coli* positive (*p* = 0.91) (Table 3). The control and intervention households used untreated water sources with similar risk for fecal contamination (*p* = 0.79) (Table 4). Following the introduction of the SODIS intervention throughout the follow-up period, the high risk for fecal contamination of drinking-water was brought to low-risk (57.9%) levels (Table 5). Water turbidity was measured on samples taken from water sources, household containers, and SODIS bottles with turbidity results of 19.3, 31.6, and 15.9 NTU on average, respectively. No chlorine residuals were observed in both the source and household water samples in the intervention and control groups during the trial time. In addition, no household reported the treatment of water with chlorine solution.

**Table 3:** Bacteriological analysis of community water supply from untreated water sources, Dabat district, northwest Ethiopia, January to June 2016

Sampling period	Results	Control (total water samples = 86) *n* (%)	Intervention (total water samples = 102) *n* (%)	*p* value
At the start of follow-up	Positive sample	43 (95.6)	51 (89.5)	0.26
At the mid follow-up	Positive sample	35 (85.4)	42 (93.3)	0.23
Overall results	Positive sample	78 (90.7)	93 (91.2)	0.91

**Table 4:** Bacteriological analysis of community water supply from untreated water sources with different risk categories in Dabat district, northwest Ethiopia, January to June 2016

Group	Number of *E. coli* (CFU/100 mL) in each risk category (*n* = 188)			*p* value
	0–10 (low risk)	11–100 (medium risk)	> 100 (high risk)	
Control, *n* (%)	16 (18.6)	21 (24.4)	49 (57.0)	0.79
Intervention, *n* (%)	23 (22.5)	26 (25.5)	53 (52.0)	

*n* number of water samples, *CFU* Coliform Forming Units

**Table 5:** Bacteriological analysis of water quality treated with Solar Disinfection (SODIS) in Dabat district, northwest Ethiopia, January to June 2016 (*n* = 57)

Sampling round	Number of *E. coli* (CFU/100 mL) with risk categories	
	0–10 (low risk)	11–100 (medium risk)
At the start of follow-up, *n* (%)	14 (77.8)	4 (22.2)
At mid follow-up, *n* (%)	19 (48.7)	20 (51.3)
Overall results, *n* (%)	33 (57.9)	24 (42.1)

### Diarrhea occurrence

Prior to the start of the SODIS intervention study, a 2-week prevalence of diarrhea was 20.8% (80/384) in the intervention and 21.8% (86/394) in the control groups as recorded during the baseline survey. There were significantly fewer cases of diarrhea, i.e., 8.3 episodes per 100 person-week observations (378 episodes of diarrhea) among U5C in the SODIS intervention group and 15.3 episodes of diarrhea per 100 person-week observations (720 episodes of diarrhea) in the control during the entire 6-month consecutive follow-up.

**Fig. 4:**
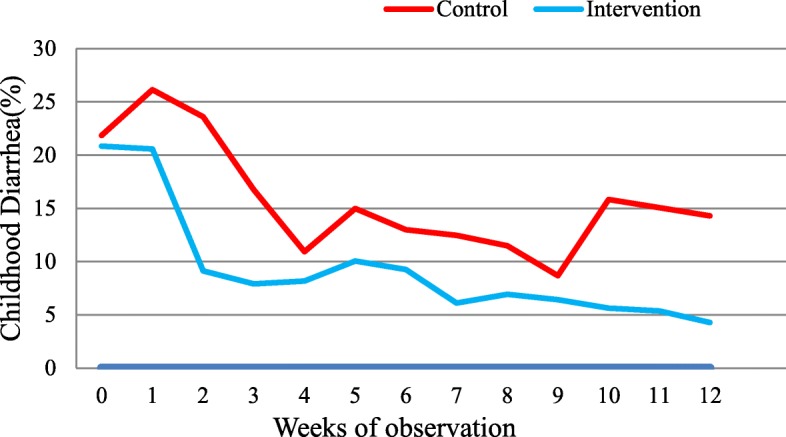
Trends of childhood diarrhea distribution during 6 months among SODIS intervention and control groups in Dabat district, northwest Ethiopia, 10 January to 7 July 2016 (0 weeks of observation = baseline survey)

The impact of the SODIS intervention varied among different age groups of U5C. The burden of diarrhea dropped by 46% and 54% among children of 2 to 3 and 3 to 5 years of age, respectively; the reduction was relatively smaller in children under 2 years of age (39%) (Table 6). The relationship between diarrhea and observation weeks in the control and intervention groups is illustrated in Fig. 4.

**Table 6:** Effect of the Solar Disinfection (SODIS) intervention within different age groups of children 6 to 59 months of age, Dabat district, northwest Ethiopia, 10 January to 7 July 2016

Age category	Control groups (*n* = 394)			Intervention group (*n* = 384)			DD reductio*n* (%)	*p* value
	Number of DD^a^ episodes	Person-week observations	DD incidence (%)	Number of DD episodes	Person-week observations	DD incide-nce (%)		
6–23 months	308	1277	24.1	183	1256	14.6	39	< 0.001
24–36 months	205	1131	18.1	102	1052	9.7	46	< 0.001
37–48 months	124	1183	10.5	60	1248	4.8	54	< 0.001
49–59months	83	1125	7.4	33	974	3.4	54	< 0.001
All < 60 months	720	4716	15.3	378	4530	8.3	46	< 0.001

^a^Diarrheal disease. The incidence of diarrhea was computed as the number of new episodes divided by the total number of person-week observations

GEE analysis showed a statistically significant reduction in the risk of diarrhea among the SODIS intervention group compared to the control group after adjusting for child age, child sex, drinking-water source, and availability of latrine. Children in the SODIS intervention group had a low risk of diarrheal incidence rate (IRR) 0.60; 95% CI (0.52, 0.70). The overall corresponding prevention of diarrheal incidence among U5C during the 6 month SODIS intervention was 40% (95% CI 30–48) compared to the control group (Table 7).

**Table 7:** Multivariable analysis of the Solar Disinfection (SODIS) intervention effects on the incidence of diarrhea among under-five children, Dabat district, northwest Ethiopia, 10 January to 7 July 2016

Factors	Crude IRR (95% CI)	Adjusted IRR (95% CI)	*p* value
Intervention	0.59 (0.51,0.70)	0.60 (0.52,0.70)	*p* < 0.001
Control	1.0	1.0	
Age of child	0.967 (0.962, 0.972)	0.966 (0.963, 0.972)	*p* < 0.001
Sex of child			
Male	0.92 (0.79, 1.09)	0.99 (0.86, 1.15)	0.934
Female	1.0	1.0	
Water source			
Improved	0.95 (0.80, 1.13)	0.99 (0.83, 1.18)	0.937
Unimproved	1.0	1.0	
Sanitation			
Not having latrine	1.08 (0.89, 1.30)	1.09 (0.92, 1.31)	0.312
Having latrine	1.0	1.0	

*IRR* incidence rate ratio

## Discussion

A community-based, cluster RCT conducted in 28 rural villages to assess the effectiveness of the SODIS method of HWT in reducing diarrhea among children under 5 years of age is the first SODIS intervention study in Ethiopia. The study arrived at a scientific evidence that SODIS as a method of HWT has a potential role as a diarrheal disease prevention strategy.

The incidence of diarrhea among U5C receiving the intervention with SODIS-treated water was found to drop compared to the control group (IRR = 0.60; 95% CI 0.52, 0.70). The corresponding reduction of diarrheal risk with those of SODIS intervention among the U5C was 40% with a 95% CI of 30–48. This finding is consistent with similar studies conducted in Kenya (44%) [53], Cambodia (50%) [54], and Cameroon (42.5%) [55]. Our finding of 40% of diarrheal reduction is higher than those of other studies in India (36%) [56], South Africa (36%) [57], and Bolivia (26%) [58]. Further, a proportion significance test with 95% CI showed a *p* value > 0.05 when compared with findings in Kenya, Cameroon, India, and South Africa and a *p* value < 0.05 when compared with those in Cambodia and Bolivia. The high magnitude of the protective effect noted in this study could be attributed to the high compliance to the intervention as observed during regular supervisions. This may be the result of the efforts of the assigned local personnel who remind households to regularly use SODIS.

The protocols of the SODIS intervention mostly followed similar characteristics as other RCTs that were conducted in Kenya [53], Cambodia [54], South Africa [57], India [56], and Bolivia [58]. The current trial used 1- and 2-L capacity transparent PET bottles, the most common container type [59], for a minimum of 6 h exposure of water to direct sunlight, and 2-weekly data collection for the primary outcomes. Most of the previous studies [32, 58–60] positively suggested that SODIS water treatment with natural sunlight is simple to use, eco-friendly, harmless to users, and the most cost-effective technology for developing countries. SODIS is cost-effective for Ethiopian rural communities as well. For instance, in the study area, a 2-L capacity transparent PET bottle can be bought at a price of ETB 6 (USD 0.28) from the local edible-oil retail shops and can serve an average family for at least 6 months. Many of the households can afford ETB 60 (USD 2.8) to buy 10 such bottles for SODIS treatment of 20 L of drinking-water daily for 6 months. Creating a supply chain, such as local market systems, for a community for the distribution of PET bottles is one of the proposed strategies to reach rural communities which rely on untreated water and are exposed to high-risk diarrheal diseases.

The SODIS interventional effect may be greater where fecal contamination of drinking-water is more likely [61]. High fecal contamination of both water sources and stored water was detected in each of the trial groups during the bacteriological analysis of water at the beginning and in the middle of the follow-up study. This could be the reason for the control group household report of a 1.84 times greater occurrence of diarrheal incidence among their children than among the children of the intervention group households (diarrheal incidence: control 15.3% vs. intervention 8.3%). Similarly, in Cameroon, regular SODIS-user households reported less diarrhea occurrence among their young children than the households of the control group [55]. These findings of this study in Ethiopia and those of all other countries prove that the SODIS intervention is effective in reducing diarrhea among children of under 5 years of age.

In the present study, the prevention of the risk of diarrhea among children under 2 years of age by SODIS water treatment was reported to be slightly less effective than among older children. This result is consistent with those of other HWT studies conducted in Guatemala [62] and eastern Ethiopia [42]. This might be due to the high susceptibility and higher chance of exposure by crawling and playing in contaminated environments in addition to weaning-time uptake of supplementary fluids prepared with untreated water [42].

The overall coverage of adherence to SODIS practice among intervention households during the 6-month follow-up study was 90.6%. This result was confirmed by the observation of field workers who evaluated the performances of households in implementing the SODIS method for HWT.

On the basis of their direct observations of the implementation of the three intervention indicators by households and in accordance with the replies of mothers/caregivers to their frequent inquiries about compliance with SODIS, the subjective judgment of field workers was that the performance of 87% of the intervention households was good. This finding of the level of compliance with SODIS in the current study was not only in line with, but also higher than, those of SODIS trials in South Africa [57] in which 75% of the participants implemented the trial protocol and Bolivia [58] wherein 80% of the intervention households used SODIS. This result might be due to the close follow-up of locally recruited field workers who live in the same villages to remind SODIS intervention households and their close supervision of the distribution of pieces of CIS for placing SODIS bottles for sunlight exposure and the good motivation of family members, especially mothers/caregivers to provide safe drinking-water to their children by using the SODIS method which is new to them.

Previous studies [60, 63] reported that the effectiveness of SODIS was affected by levels of turbidity and seasonal variations. Most of the drinking-water sources were characterized by threshold turbidity levels for SODIS efficiency in the dry season (i.e., < 30 NTU) [59, 64]. In the present study, the turbidity of treatable water was found to be low (average turbidity 15.9 NTU). This was because the study was conducted in the Ethiopian dry season. In some cases, there can be a visible turbidity in open water sources. Households were well informed to clear the water by using plain sedimentation and filtration by cloth or other local sieves before filling the SODIS bottle by hand.

### Limitations

The invisible and unavoidable problem encountered was the difficulty in preventing children from exposure to other transmission routes of infection, like drinking-water from untreated water sources, and for fear of bottle security, some of the mothers/caregivers went to market without exposing SODIS bottles to sunlight. The SODIS intervention might, therefore, be associated with courtesy- and participant-biased reporting as a result of the open-label nature of the trials [65] through the visible display of bottles to sunlight in the villages. Similar problems are common to nearly all HWT interventions [66]. Schmidt and Cairncross [67] argue that reporting bias has been the dominant problem in non-blinded studies. Indeed, the ultimate goal of the trial was not explained to household members and field workers. In fact, the duty of the field workers was to introduce bottles for the SODIS intervention to households and to collect data without the interference of the principal investigator. Mausezahl and colleagues indicate that using independent data collectors and intervention implementers in the absence of blinding is critical in reducing bias in home-based SODIS trials [58]. The other limitation of this study was that the outcome data (diarrhea) were collected based on mothers’/ caregivers’ self-reports. This may contribute to a somehow inconsistent estimation of observed results. But attempts were made to minimize the effect of respondent recall bias through cross-checking the daily filled pictorial morbidity diaries from each household.

## Conclusion

In conclusion, the SODIS intervention substantially reduced the incidence of diarrhea among U5C in the rural community of northwest Ethipia, where fecal contamination of drinking-water was high. SODIS can be seen as a new potential HWT alternative strategy for rural Ethiopia to prevent/reduce the risk of child morbidity and mortality caused by diarrhea toll. This significantly contributes to the achievement of sustanaible development goals that dealt with improved child health and increased safe water access. In general, communities with scarcity of safe water in other sub-Saharan countries can also implement the SODIS water treatment intervention to prevent waterborne diarrheal diseases. Therefore, the SODIS intervention is an invaluable strategy to be integrated with national health extension programs to address rural communities.

## Additional files

Additional file 1: Protocol S1: Trial protocol. (DOCX 31 kb)

Additional file 2: Checklist S2: Consolidated Standards of Reporting Trials (CONSORT) 2010 Checklist. (DOC 217 kb)

## Abbreviations

EDHS: Ethiopia Demographic and Health Surveillance
GEE: Generalized estimating equation
HDSS: Health and Demographic Surveillance System
HWT: Household water treatment
ICC: Intracluster coefficient
PET: Polyethylene terephthalate
RCT: Randomized controlled trial
SODIS: Solar water disinfection
U5C: Under-five children
UNICEF: United Nation International Children’s Emmergency Fund
WHO: World Health Organization
