# Supplementary material for: The effect of SODIS water treatment intervention at the household level in reducing diarrheal incidence among children under 5 years of age: a cluster randomized controlled trial in Dabat district, northwest Ethiopia
Source: Trials. 2018 Jul 31;19:412. doi: 10.1186/s13063-018-2797-y (PMC6069566; doi:10.1186/s13063-018-2797-y)
Supplement: Supplementary file 1 — Protocol S1: Trial protocol. (DOCX 31 kb) [file 13063_2018_2797_MOESM1_ESM.docx]

| **FULL DETAILS (Read-only)  ->**[**Click Here to Create PDF for Current Dataset of Trial**](http://ctri.nic.in/Clinicaltrials/pdf_generate.php?trialid=20038&EncHid=46059.26420&modid=1&compid=19','20038det') |
| --- |

| **CTRI No** | **CTRI/2017/09/009640** [Registered on: 05/09/2017] **Trial Registered Retrospectively** |
| --- | --- |
| **Acknowledgement Number** | REF/2017/08/015162 |
| **Last Modified On:** | 04/09/2017 |
| **Post Graduate Thesis** | Yes |
| **Type of Trial** | Interventional |
| **Type of Study** | Preventive |
| **Study Design** | Cluster Randomized Trial |
| **Public Title of Study**  [Clarification(s) with Reply](javascript:newwin2('viewmod.php?trialid=20038&blid=1&EncHid=46059.26420&modid=1&compid=19','20038cla'))  [Modification(s)](javascript:newwin2('viewmod.php?trialid=20038&blid=1&EncHid=46059.26420&modid=1&compid=19','20038modi')) | SODIS Water Treatment Intervention to reduce childhood diarrhea:A Clustered Randomized Controlled Trial in Northwest Ethiopia |
| **Scientific Title of Study**  [Clarification(s) with Reply](javascript:newwin2('viewmod.php?trialid=20038&blid=2&EncHid=46059.26420&modid=1&compid=19','20038cla'))  [Modification(s)](javascript:newwin2('viewmod.php?trialid=20038&blid=2&EncHid=46059.26420&modid=1&compid=19','20038modi')) | Household water treatment by solar disinfection as a method of diarrhoeal disease prevention among under five children in Dabat district, northwest Ethiopia |
| **Acronym** | SODIS-HWT |
| **Secondary IDs if Any** | \| **Secondary ID** \| **Identifier** \| \| --- \| --- \| \| N/A \| NIL \| |
| **Details of Principal Investigator or overall Trial Coordinator (multi-center study)** | \| **Name** \| Bikes Destaw Bitew \| \| --- \| --- \| \| **Designation** \| Assistant professor \| \| **Affliation** \| University of Gondar \| \| **Address** \| University of Gondar, College of Medicine and Health Sciences, Institute of Public Health, Ethiopia Azezo   196 Other \| \| **Phone** \| 918785876 \| \| **Fax** \|  \| \| **Email** \| bikesdestaw2004@gmail.com \| |
| **Details Contact Person Scientific Query** | \| **Name** \| Gashaw Andargie Biks \| \| --- \| --- \| \| **Designation** \| Associate Professor \| \| **Affliation** \| University of Gondar \| \| **Address** \| Institute of Public Health, College of Medicine and Health Sciences, University of Gondar, Ethiopia Gondar   196 Other \| \| **Phone** \| 913385423 \| \| **Fax** \| 581116221 \| \| **Email** \| gashawab@gmail.com \| |
| **Details Contact Person Public Query** | \| **Name** \| Yigzaw Kebede Gete \| \| --- \| --- \| \| **Designation** \| Professor \| \| **Affliation** \| University of Gondar \| \| **Address** \| Institute of Public Health, College of Medicine and Health Sciences, University of Gondar, Ethiopia Gondar   196 Other \| \| **Phone** \| 911522831 \| \| **Fax** \| 581116221 \| \| **Email** \| gkyigzaw@yahoo.com \| |
| **Source of Monetary or Material Support**  [Clarification(s) with Reply](javascript:newwin2('viewmod.php?trialid=20038&blid=7&EncHid=46059.26420&modid=1&compid=19','20038cla'))  [Modification(s)](javascript:newwin2('viewmod.php?trialid=20038&blid=7&EncHid=46059.26420&modid=1&compid=19','20038modi')) | \| University of Gondar, Vice president for Research and community service. Email:kasuamare@gmail.com Phone:581141236 P.O.Box:196 Gondar, Ethiopia \| \| --- \| |
| **Primary Sponsor** | \| **Name** \| University of Gondar \| \| --- \| --- \| \| **Address** \| Gondar, Ethiopia \| \| **Type of Sponsor** \| Other [Government University ] \| |
| **Details of Secondary Sponsor** | \| **Name** \| **Address** \| \| --- \| --- \| \| NA \| NA \| |
| **Countries of Recruitment** | Ethiopia |
| **Sites of Study**  [Clarification(s) with Reply](javascript:newwin2('viewmod.php?trialid=20038&blid=11&EncHid=46059.26420&modid=1&compid=19','20038cla'))  [Modification(s)](javascript:newwin2('viewmod.php?trialid=20038&blid=11&EncHid=46059.26420&modid=1&compid=19','20038modi')) | \| No of Sites = **1** \| \| \| \| \| --- \| --- \| --- \| --- \| \| **Name of Principal Investigator** \| **Name of Site** \| **Site Address** \| **Phone/Fax/Email** \| \| Takele Tadesse Adafirie \| Dabat DHSS site, in 28Villages, Dabat district, Northwest Ethiopia \| Dabat research Center, Dabat District, North Gondar Administrative Zone, Amhara Administrative Regional Sate, Northwest Ethiopia P.O.Box. 196 \| 920256715  takeletadesse1627@gmail.com \| |
| **Details of Ethics Committee** | \| No of Ethics Committees= **1** \| \| \| \| \| \| --- \| --- \| --- \| --- \| --- \| \| **Name of Committee** \| **Approval Status** \| **Date of Approval** \| **Approval Document** \| **Is IEC?** \| \| Institutional Ethical Review Board of University of Gondar \| Approved \| 17/04/2015 \| [Approval File](http://ctri.nic.in/Clinicaltrials/WriteReadData/ethic/4184944920EthicalclearancebyUoG.jpg) \| Yes \| |
| **Regulatory Clearance Status from DCGI** | \| **Status** \| **Date** \| **Aproval Document** \| \| --- \| --- \| --- \| \| Not Applicable \| No Date Specified \| No File Uploaded \| |
| **Health Condition / Problems Studied** | \| **Health Type** \| **Condition** \| \| --- \| --- \| \| Healthy Human Volunteers \| at the base line they were healthy. \| |
| **Intervention / Comparator Agent** | \| **Type** \| **Name** \| **Details** \| \| --- \| --- \| --- \| \| Comparator Agent \| Control group: Non SODIS water treatment user \| Households with under five children in the 14 clusters(Villages) enrolled for non SODIS user for drinking water treatment for consecutive 6months. \| \| Intervention \| SODIS water treatment intervention \| Households with under five children in the 14 clusters(Villages) enrolled for household water treatment intervention group for consecutive 6months. \| |
| **Inclusion Criteria**  [Clarification(s) with Reply](javascript:newwin2('viewmod.php?trialid=20038&blid=17&EncHid=46059.26420&modid=1&compid=19','20038cla'))  [Modification(s)](javascript:newwin2('viewmod.php?trialid=20038&blid=17&EncHid=46059.26420&modid=1&compid=19','20038modi')) | \| **Age From** \| 6.00 Month(s) \| \| --- \| --- \| \| **Age To** \| 59.00 Month(s) \| \| **Gender** \| Both \| \| **Details** \| Inclusion criteria:(i) geographical accessibility of a cluster area throughout the year; (ii) the average size of cluster was 28-30 children under five years; (iii) reliance only on untreated drinking water sources and (iv) no other special water quality management intervention \| |
| **Exclusion Criteria** | \| **Details** \| Exclusion criteria of Participants:  Children less than 6 months of age, and children who were received special diarrhea prevention/control programs in the study area. \| \| --- \| --- \| |
| **Method of Generating Random Sequence** | Coin toss, Lottery, toss of dice, shuffling cards etc |
| **Method of Concealment** | Centralized |
| **Blinding/Masking** | Not Applicable |
| **Primary Outcome**  [Clarification(s) with Reply](javascript:newwin2('viewmod.php?trialid=20038&blid=21&EncHid=46059.26420&modid=1&compid=19','20038cla'))  [Modification(s)](javascript:newwin2('viewmod.php?trialid=20038&blid=21&EncHid=46059.26420&modid=1&compid=19','20038modi')) | \| **Outcome** \| **TimePoints** \| \| --- \| --- \| \| Childhood diarrhoea incidence \| Data assessed biweekly bases: week1:24/01/2016  week2:08/02/2016 week3:23/02/2016 week4:09/03/2016 week5:24/03/2016 week6:08/04/2016 week7:23/04/2016 week8:08/05/2016 Week9:23/05/2016 week10:07/06/2016 week11:22/06/2016 week12:07/07/2016 \| |
| **Secondary Outcome**  [Clarification(s) with Reply](javascript:newwin2('viewmod.php?trialid=20038&blid=22&EncHid=46059.26420&modid=1&compid=19','20038cla'))  [Modification(s)](javascript:newwin2('viewmod.php?trialid=20038&blid=22&EncHid=46059.26420&modid=1&compid=19','20038modi')) | \| **Outcome** \| **Time Points** \| \| --- \| --- \| \| Not applicable \| Not applicable \| |
| **Target Sample Size**  [Clarification(s) with Reply](javascript:newwin2('viewmod.php?trialid=20038&blid=23&EncHid=46059.26420&modid=1&compid=19','20038cla'))  [Modification(s)](javascript:newwin2('viewmod.php?trialid=20038&blid=23&EncHid=46059.26420&modid=1&compid=19','20038modi')) | **Total Sample Size=**"797" **Sample Size from India=**"0"  **Final Enrollment numbers achieved (Total)=** "766"  **Final Enrollment numbers achieved (India)=**"0" |
| **Phase of Trial** | N/A |
| **Date of First Enrollment (India)**  [Clarification(s) with Reply](javascript:newwin2('viewmod.php?trialid=20038&blid=25&EncHid=46059.26420&modid=1&compid=19','20038cla'))  [Modification(s)](javascript:newwin2('viewmod.php?trialid=20038&blid=25&EncHid=46059.26420&modid=1&compid=19','20038modi')) | No Date Specified |
| **Date of Study Completion (India)** | Date Missing |
| **Date of First Enrollment (Global)** | 10/01/2016 |
| **Date of Study Completion (Global)** | 07/07/2016 |
| **Estimated Duration of Trial** | **Years=**"0" **Months=**"6" **Days=**"0" |
| **Recruitment Status of Trial (Global)**  [Clarification(s) with Reply](javascript:newwin2('viewmod.php?trialid=20038&blid=27&EncHid=46059.26420&modid=1&compid=19','20038cla'))  [Modification(s)](javascript:newwin2('viewmod.php?trialid=20038&blid=27&EncHid=46059.26420&modid=1&compid=19','20038modi')) | Completed |
| **Recruitment Status of Trial (India)** | Completed |
| **Publication Details**  [Clarification(s) with Reply](javascript:newwin2('viewmod.php?trialid=20038&blid=29&EncHid=46059.26420&modid=1&compid=19','20038cla'))  [Modification(s)](javascript:newwin2('viewmod.php?trialid=20038&blid=29&EncHid=46059.26420&modid=1&compid=19','20038modi')) | du Preez M, Conroy RM, Ligondo S, Hennessy J, Elmore-Meegan M, Soita A, McGuigan KG: Randomized intervention study of solar disinfection of drinking water in the prevention of dysentery in Kenyan children aged under 5 years. Environmental science & technology 2011, 45(21):9315-9323. McGuigan KG, Samaiyar P, du Preez M, Conroy RM: High compliance randomized controlled field trial of solar disinfection of drinking water and its impact on childhood diarrhea in rural Cambodia. Environmental science & technology 2011, 45(18):7862-7867. Mausezahl D, Christen A, Pacheco GD, Tellez FA, Iriarte M, Zapata ME, Cevallos M, Hattendorf J, Cattaneo MD, Arnold B: Solar drinking water disinfection (SODIS) to reduce childhood diarrhoea in rural Bolivia: a cluster-randomized, controlled trial. PLoS medicine 2009, 6(8):882. |
| **Brief Summary** | The magnitude of diarrhoeal disease is high in developing countries where an estimated 801,000 children under five die due to diarrhea each year. Consumption of water from unimproved sources is a potential contributor of diarrhoeal diseases and their transmission. In sub-Saharan Africa, safe water coverage is less than 50% where about 319 million people lack access to improved water sources and an estimated 1.9 billion people rely on faecally contaminated drinking-water.   In Ethiopia, nearly 40 million people, most of them in rural areas, didn’t have access to safe drinking water. Even water safe at the source can be easily contaminated during collection, transport, and storage. However; more than 90% of households do not treat their drinking water at home. Such a situation would pose high public health risks of diarrhea to unsafe water users unless prompt intervention is implemented. Evidences revealed in different parts of Ethiopia indicated that the two-week period prevalence of diarrhea among under-five children ranged from 22.5 to 30.5%.  Installation of large scale water treatment plants in rural Ethiopia is difficult due to the scarcity of resources including poor infrastructure Therefore, the situation demands implementation of alternative strategies such as easily applicable low-cost and environmental friendly household water treatment such as solar water disinfection (SODIS). Solar water disinfection (SODIS) where raw water is filled in polyethylene terephthalate (PET) bottles is one of the potential alternative household water treatment technologies that rely on the germicidal effects of sunlight and heat. However, SODIS is still largely unknown as a method of household water treatment technology in rural Ethiopia. So, evidence based health effect of SODIS intervention at household level is limited. The main purpose of this study is to examine the effect of SODIS intervention in reducing the burden of diarrhoeal disease among under-five children in rural community of northwest Ethiopia.  A community based clustered randomized controlled trial was conducted among children under five years of age in each community from January 10 to July 7, 2016. The SODIS intervention was designed according to the Swiss Federal Institute for Environmental Science and Technology (EWAG) published guideline. Initially, the study area was divided into two blocks (“block A” and “block B”) with an adequate buffering zone between them. Potential clusters in the study area were exhaustively listed and sorted out the eligible clusters based on the preset selection criteria within the two blocks. Intervention and control blocks/arms were randomly assigned through lottery systems. Clusters were also selected in each block based on lottery methods. In the eligible 28 clusters (villages), households with under-five children were randomly allocated to the intervention and control groups and then assigned within each of the 14 pairs of communities randomly to one of them. In each cluster, 28-30 children (778 in total) were enrolled within 568 households and followed up for 6 consecutive months. Nineteen data collectors with 6 supervisors approached the mother-child pairs in the selected households and completed the baseline survey with regard to socio-demographic, environmental, and behavioral characteristics of each household in the community and follow up study.  The primary outcome of the study is childhood diarrhea which is defined by WHO as “three or more loose or watery stools over the past 24 hours (or more frequently than it is normal for the individual). It was computed as the number of under five children (U5C) who are new cases of a diarrhoeal disease over a six months of follow up period divided into person-weeks observation at risk during the similar period of time. Data were collected biweekly bases.  This research is operated under the aegis of Institute of Public Health, College of Medicine and Health Sciences, University of Gondar, Ethiopia.   **Clinical trial protocol registry system is not available in Ethiopia.** |
